# CNN-based CP-OCT sensor integrated with a subretinal injector for retinal boundary tracking and injection guidance

**DOI:** 10.1117/1.JBO.26.6.068001

**Published:** 2021-06-30

**Authors:** Soohyun Lee, Jin U. Kang

**Affiliations:** Johns Hopkins University, Department of Electrical and Computer Engineering, Baltimore, Maryland, United States

**Keywords:** optical coherence tomography, convolutional neural network, retinal segmentation, microsurgery, surgical guidance

## Abstract

**Significance:** Subretinal injection is an effective way of delivering transplant genes and cells to treat many degenerative retinal diseases. However, the technique requires high-dexterity and microscale precision of experienced surgeons, who have to overcome the physiological hand tremor and limited visualization of the subretinal space.

**Aim:** To automatically guide the axial motion of microsurgical tools (i.e., a subretinal injector) with microscale precision in real time using a fiber-optic common-path swept-source optical coherence tomography distal sensor.

**Approach:** We propose, implement, and study real-time retinal boundary tracking of A-scan optical coherence tomography (OCT) images using a convolutional neural network (CNN) for automatic depth targeting of a selected retinal boundary for accurate subretinal injection guidance. A simplified 1D U-net is used for the retinal layer segmentation on A-scan OCT images. A Kalman filter, combining retinal boundary position measurement by CNN and velocity measurement by cross correlation between consecutive A-scan images, is applied to optimally estimate the retinal boundary position. Unwanted axial motions of the surgical tools are compensated by a piezoelectric linear motor based on the retinal boundary tracking.

**Results:** CNN-based segmentation on A-scan OCT images achieves the mean unsigned error (MUE) of ∼3  pixels (8.1  μm) using an *ex vivo* bovine retina model. GPU parallel computing allows real-time inference (∼2  ms) and thus real-time retinal boundary tracking. Involuntary tremors, which include low-frequency draft in hundreds of micrometers and physiological tremors in tens of micrometers, are compensated effectively. The standard deviations of photoreceptor (PR) and choroid (CH) boundary positions get as low as 10.8  μm when the depth targeting is activated.

**Conclusions:** A CNN-based common-path OCT distal sensor successfully tracks retinal boundaries, especially the PR/CH boundary for subretinal injection, and automatically guides the tooltip’s axial position in real time. The microscale depth targeting accuracy of our system shows its promising possibility for clinical application.

## 1 Introduction

Subretinal injection is becoming increasingly prevalent in both scientific research and clinical communities as an efficient way of treating retinal diseases. It has been used for gene and cell transplant therapies to treat many degenerative vitreoretinal diseases, such as retinitis pigmentosa, age-related macular degeneration, and Leber’s congenital amaurosis.^1^ The treatments involve the delivery of drugs or stem cells into subretinal space between the RPE and photoreceptor (PR) layer, thereby directly affecting resident cells and tissues in the subretinal space. However, the procedure requires surgeons’ high-dexterity and microscale precision due to the delicate anatomy of the retina. The procedure is further complicated by the existence of physiological motions by patients, surgeons’ hand tremor^2^^,^^3^ and limited depth perception, and limited visual feedback from a traditional stereo-microscopic *en face* view.

Optical coherence tomography (OCT)-guided robotic systems have been developed to reduce the unintended physiological motion and overcome the limited visual feedback during ocular microsurgery. OCT, which provides microscale resolution cross-sectional images in real time,^4^ enables improved visualization and accurate guidance of robotic systems. Microscope-integrated OCT systems were applied for surgical tool localization and robotic system guidance by intraoperatively providing volumetric images of tissues and surgical tools.^5^^–^^9^ Fiber-optic common-path OCT (CP-OCT) distal sensor integrated hand held surgical devices have also been developed to implement simple, compact, and cost-effective microsurgical systems.^10^^–^^13^ In those systems, a single-fiber distal sensor attached to a surgical tooltip (i.e., needle or microforceps) guided the hand held surgical device by real-time A-scan-based surface tracking. However, surface tracking-based guidance could induce inaccurate depth targeting for subretinal injection because of retinal thickness variations and irregular morphological features caused by retinal diseases. The target or near-target retinal boundary tracking, which is the RPE and PR boundary tracking for subretinal injection, allows precision guidance, but previous researches on retinal layer segmentation of OCT images using active contours,^14^^,^^15^ graph search,^16^^–^^18^ and shortest path methods^19^^,^^20^ are not adequate for A-scan images due to the absence of lateral information. In recent years, convolutional neural network (CNN)-based retinal layer segmentation have been proposed and showed promising results.^21^^–^^24^ Although the proposed CNN-based methods were developed for B-scan or C-scan OCT image segmentation, they could also be applied to A-scan images and operate in real time by simplifying networks and using GPU parallel computing.

In this paper, we present real-time retinal boundary tracking based on CNN segmentation of A-scan OCT images for accurate depth targeting of a selected retinal boundary. The U-net,^25^ which is widely used in medical image segmentation, was simplified and applied for segmentation on A-scan images. A Kalman filter, combining retinal boundary position measurement by CNN and velocity measurement by cross correlation between consecutive A-scan images, is applied to optimally estimate the retinal boundary position. Undesired axial motions of the surgical tool are compensated by a piezoelectric linear motor using the tracked boundary position. An *ex vivo* bovine eye model is used to evaluate the retinal boundary tracking and depth targeting performance of the hand held microsurgical device.

## 2 Experiments and Methods

### 2.1 Network Architecture and Training for Retinal Layer Segmentation

We applied a simplified 1D U-net for A-scan retinal OCT image segmentation. The U-net is a fully CNN consisting of a contracting path to capture context followed by a symmetric expanding path that enables precise localization. In our design, double convolutional layers of the original U-net were reduced to a single convolutional layer, and the identical number of feature channels was used for all convolutional layers.

**Fig. 1:**
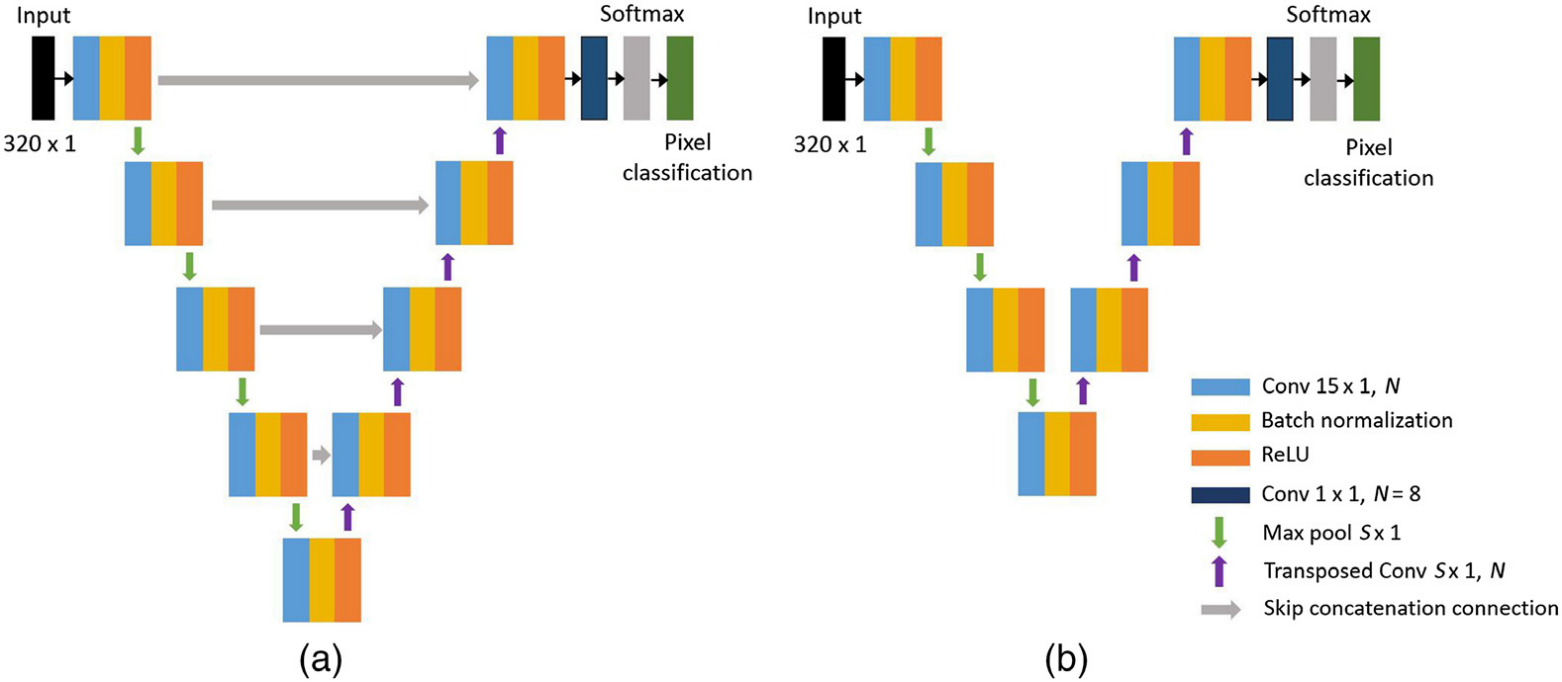
Network architecture of (a) our 1D U-net and (b) the most simplified 1D U-net we applied. N, kernel number and S, kernel size.

Figure 1(a) shows the 1D U-net architecture we designed. The contracting path is composed of four contracting blocks containing a convolutional layer, batch normalization layer, ReLU activation layer, and max-pooling layer in sequence. Similarly, the expanding path is composed of four expanding blocks containing a transposed convolution layer, concatenation layer, convolutional layer, batch normalization layer, and ReLU activation layer in sequence. The convolutional kernel size of 15×1 was used to ensure the receptive field to be larger than the image size. The receptive field is expressed as^26^

$$r=s^{b}(1+2(k-1))-k,$$ (1)

where s is the sampling size, which equals the kernel size of max-pooling layer and the transposed convolutional layer, b is the number of contracting blocks, and k is the convolutional kernel size. The kernel size of the max-pooling layer and the transposed convolutional layer was set to 2×1, and, in this case, the receptive field is calculated as 450×1. Since improving inference speed is important for our application, the 1D U-net illustrated in Fig. 1(a) was simplified stepwise, and the performance of four architectures was compared. The number of contracting and expanding blocks was reduced to three while keeping other conditions the same, and also max-pooling and transposed convolutional kernels were sized up to 4×1 for compensating reduced receptive field. We then removed skip concatenation layers to see the effect of the skip connections, and the simplest 1D U-net is illustrated in Fig. 1(b).

The 1D U-net models were implemented using Pytorch on a computer with Intel i9-10900X CPU, NVIDIA Quadro RTX 4000 GPU, and 32 GB RAM for training. A generalized dice loss function was used, and the network parameters were updated via backpropagation and Adam optimization process. Max epoch was 20, and the mini-batch size was 128. The learning rate was initialized as $10^{-3}$, which then decreases by 10 times after 10 epochs.

The trained CNN model was implemented on CUDA by customized CUDA kernels, and the inference time of the CNN models on GPU was measured using the NVIDIA Nsight tool in Visual Studio on the workstation described in Sec. 2.4.

### 2.2 Retinal Boundary Tracking

The axial distance between a fiber (needle) end and a target boundary can be measured from the target boundary position at A-scan images since the fiber end, working as a reference reflector, locates at the top edge of the images. A target boundary position was measured from a segmented image by averaging the bottommost pixel position of an adjacent upper layer and the topmost pixel position of an adjacent lower layer. Then the Kalman filter^27^ was applied to estimate the boundary position optimally using the dynamic and measurement model described as

$$x_{k}=[\begin{array}{c} x_{k}\\ v_{k} \end{array}]=Fx_{k-1}+Bu_{k-1}+w_{k}=[\begin{array}{cc} 1 & \Delta t\\ 0 & 1 \end{array}]x_{k-1}+[\begin{array}{c} 1\\ 0 \end{array}]u_{k-1}+[\begin{array}{c} \frac{1}{2}\Delta t^{2}\\ \Delta t \end{array}]a_{k},$$ (2)

$$z_{k}=Hx_{k}+n_{k}=[\begin{array}{cc} 1 & 0\\ 0 & 1 \end{array}]x_{k}+n_{k},$$ (3)

where $x_{k}$, $v_{k}$, and $a_{k}$ are the axial position, velocity, and acceleration of the target boundary. The control of the linear motor $u_{k}$ is a distance that the linear motor moves forward or backward. The velocity $v_{k}$ was measured by the ratio of movement distance of the sample (i.e., target boundary) to a known constant time duration. The movement distance was calculated by displacement of the sample in two consecutive A-scan images, which is the shift value maximizing cross correlation between two consecutive A-scan images, subtracted by the previous control $u_{k-1}$. The $u_{k}$ was defined as c ($x_{\text{target}}-x_{k}$) using proportional control, where ($x_{\text{target}}-x_{k}$) is an error and c is a proportional gain. The bias for control was set to zero because the linear motor is supposed to be stationary when the boundary position is at the target position. The proportional gain c was determined experimentally. The $w_{k}$ and $n_{k}$ are the process noise and observation noise, respectively, and they were assumed to be zero-mean Gaussian white noise. The algorithm works in two distinctive processes and is given by

prediction:

x^k|k−1=Fxk−1|k−1+Buk−1, (4)

$$P_{k|k-1}=FP_{k-1|k-1}F^{T}+Q,$$ (5)

correction:

x^k|k=x^k|k−1+Kk(zk−Hkx^k|k−1), (6)

$$K_{k}=P_{k|k-1}+P_{k|k-1}H^{T}{\left(HP_{k|k-1}H^{T}+R_{k}\right)}^{-1},$$ (7)

$$P_{k|k}=(I-K_{k}H)P_{k|k-1},$$ (8)

where P, Q, and R are the covariance of error, process noise, and observation noise, and K is the Kalman gain.

The quantitative evaluation of the retinal layer tracking performance was based on three metrics: mean signed error (MSE), mean unsigned error (MUE), and absolute maximum error (AME) of each retinal boundary position.

### 2.3 Dataset

**Fig. 2:**
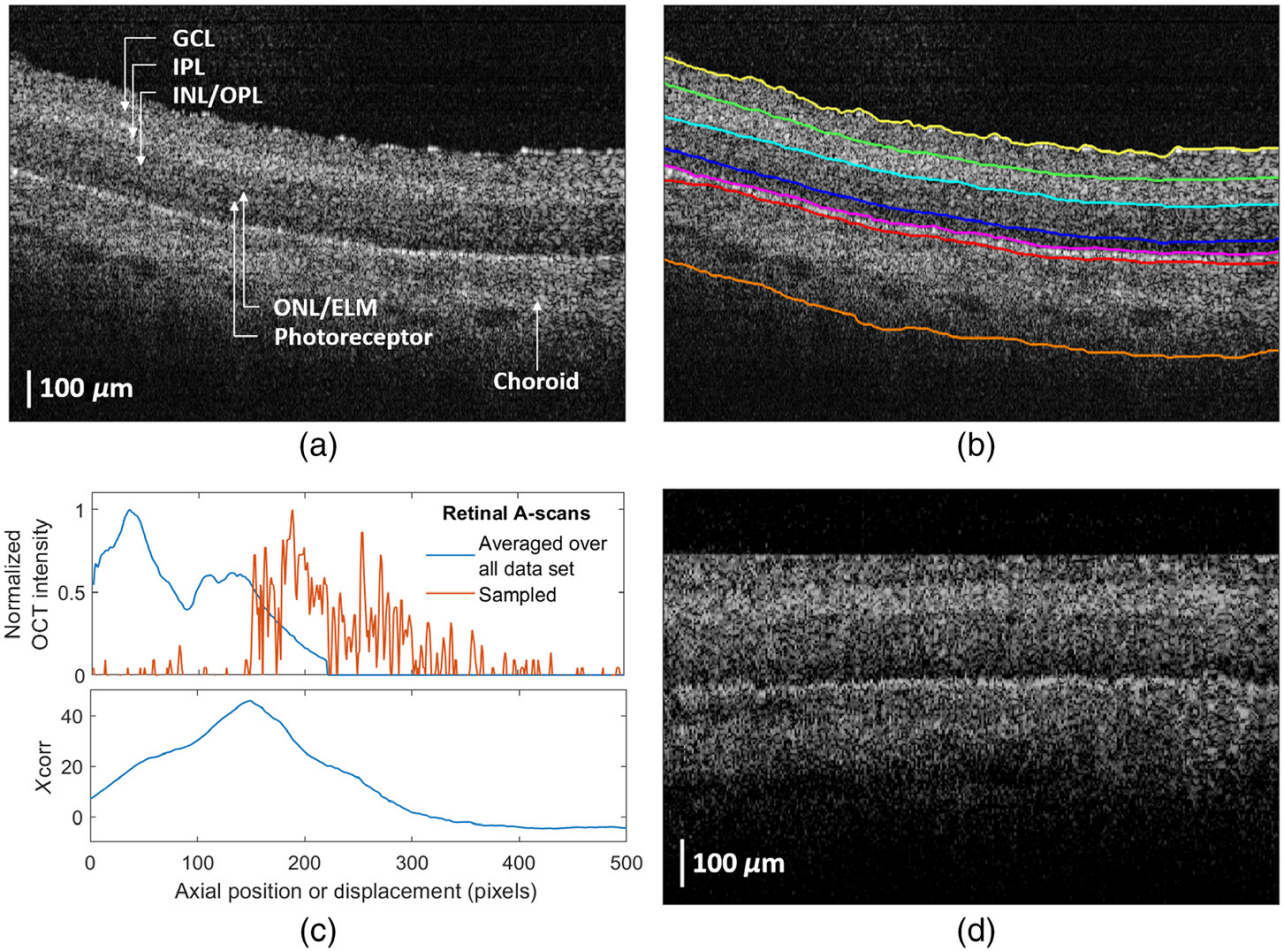
(a) A quasi B-scan OCT image of an *ex vivo* bovine eye obtained using an endoscopic CP-OCT-lensed fiber probe. (b) A manually segmented OCT image. (c) The averaged retinal A-scan over all datasets and a sampled retinal A-scan (upper graph) and cross correlation between the two A-scans (lower graph). (d) A cropped quasi B-scan OCT image consisting of the cropped A-scan images in the train dataset.

A-scan OCT images of the retina were obtained from 11 *ex vivo* bovine eyes using endoscopic CP-OCT-lensed fiber probes.^28^ The cornea and lens of the eyes were removed, and the lensed fiber probes were inserted into the vitreous humor (VH) and horizontally scanned by a motorized linear translation stage (Z812B, Thorlabs, USA). More details about the CP-OCT system are described in Sec. 2.4. Eight A-scan images were averaged to improve the signal-to-noise ratio. The resultant A-scan images were combined to present a quasi-B-scan image for easy visualization as shown in Fig. 2(a). The quasi-B-scan images were then manually segmented into the VH, the six retinal layers, labeled as ganglion cell layer (GCL), inner plexiform layer (IPL), inner nuclear layer (INL)-outer plexiform layer (OPL), outer nuclear layer (ONL)-external limiting membrane (ELM), PR layers, and choroid (CH), and region below the retina by a single observer using ImageJ software. Figure 2(b) shows the manually segmented image. 8400 A-scan OCT retinal images from 9 eyes were used for training, and 1000 A-scan OCT retinal images from 2 eyes were used for testing.

A-scan images of 1×1024  pixels were cropped into 1×320  pixels along the axial direction, keeping only the region around retinal tissues, to reduce computation time. The retinal tissue area was found using cross correlation between the averaged A-scan image over all datasets and each A-scan image. All A-scan images in the dataset were averaged, after being shifted such that the retinal layer surface lays on zero position, and then thresholded to remove background noise. The upper graph of Fig. 2(c) shows the averaged A-scan image and a sampled A-scan image from Fig. 2(a), and the lower graph shows cross correlation between the two A-scans as a function of displacement. Since the retinal surface position of the averaged A-scan is set to zero, the displacement maximizing the cross correlation indicates approximately the retinal surface location of each A-scan image. Figure 2(d) shows the cropped image obtained from Fig. 2(a).

The cropped images of the train dataset were augmented by random vertical translation. For each A-scan image, five additional training samples were created with random translation values between −15 and 15. The final train and test datasets consist of 46,530 A-scan images and 1000 A-scan images, respectively. The image pixel size along the axial direction is 2.7  μm.

### 2.4 CP-SSOCT Distal Sensor Guided Handheld Microsurgical Tool System

**Fig. 3:**
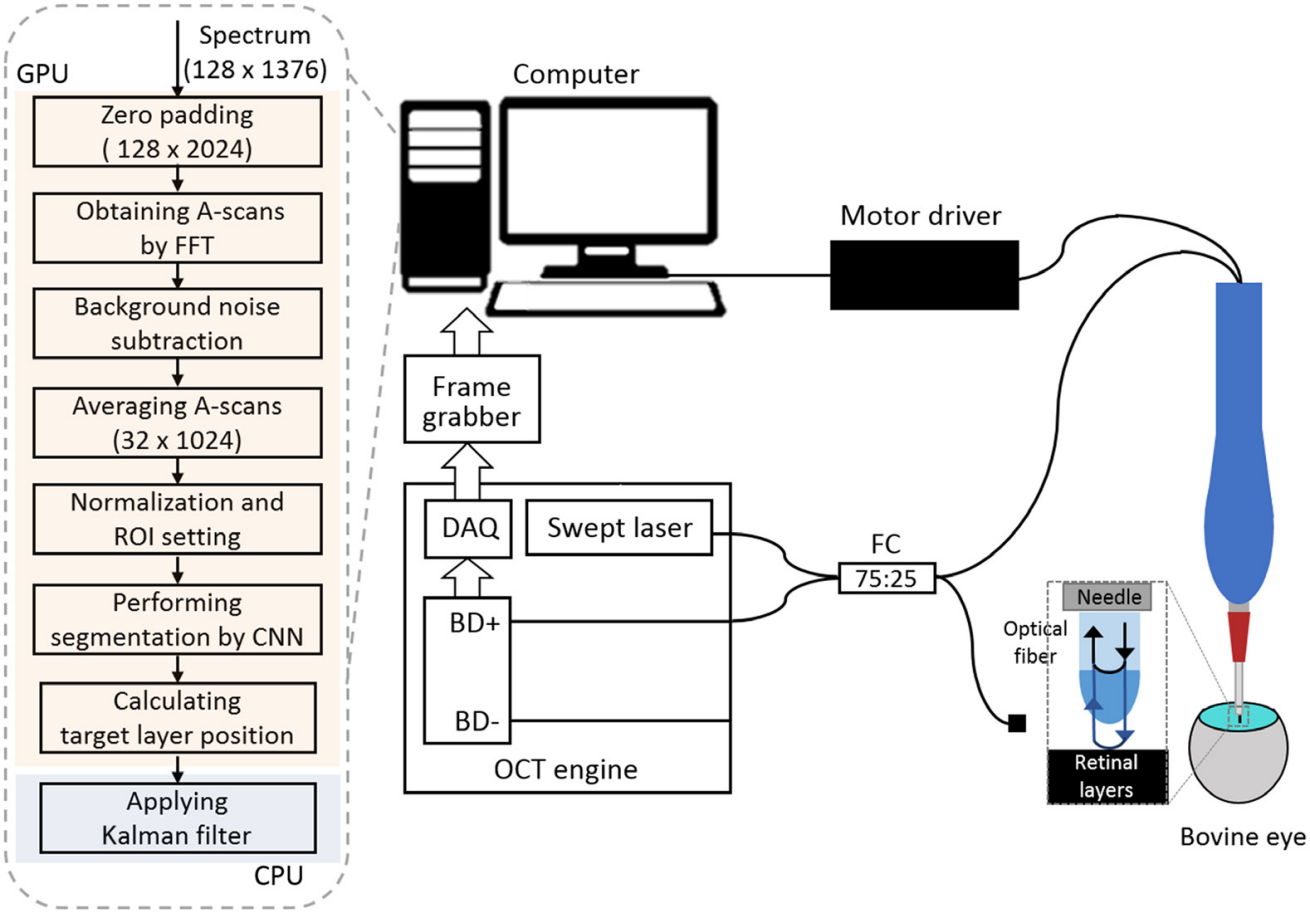
Schematic of CP-SSOCT distal sensor guided handheld microsurgical tool system and a signal processing flowchart.

Figure 3 shows the schematic of the common-path swept-source optical coherence tomography (CP-SSOCT) distal sensor-guided handheld microsurgical tool system and a signal processing flowchart. The CP-SSOCT system uses a commercial swept-source engine (Axsun Technologies Inc., Billerica, USA) operating at a 100-kHz sweep rate. The center wavelength and sweeping bandwidth of the system are 1060 and 100 nm, respectively. A lensed fiber probe of the CP-SSOCT system is encased in a 25-gauge blunt needle and fixed along the needle using UV curable glue. The fiber probe guides the needle to maintain a specified distance from a target boundary using a piezoelectric linear motor (LEGS LT20, PiezoMotor, Uppsala, Sweden). The linear motor velocity can be set as high as 12.5  mm/s, and it limits the velocity of motion it can compensate. More details about the microsurgical tool system are described in Ref. 11. A workstation (Dell Precision T5810) with an NVIDIA Quadro K4200 GPU processes the sampled spectral data to measure a distance between a target boundary and a needle and controls the linear motor. Most parts of the signal processing including CNN inference are performed on GPU by CUDA to reduce processing time. Specifically, 128 spectra were transmitted from a frame grabber and processed at the same time. A-scan images were obtained by performing the fast Fourier transform on the spectral data. After background noise subtraction, eight sequential A-scan images were averaged to increase the signal-to-noise ratio and cropped into 16×320  pixels. CNN-based segmentation is performed on the 16 cropped images of 1×320  pixels, and a target boundary distance is measured as described in Sec. 2.2. The Kalman filter is applied using the measured position and velocity, and the optimally estimated position was used for motor control.

## 3 Experimental Results and Discussion

### 3.1 Train and Test Results of CNN-Based Segmentation and Boundary Tracking

The CNN-based retinal layer segmentation performance was evaluated by mean intersection over union (IoU). The mean IoU is calculated by averaging the IoU score of each class as follows:

$$\text{mean }\mathrm{IoU}=\overset{C}{\underset{c=1}{\sum }}\frac{n_{c,\mathrm{TP}}}{n_{c,\mathrm{TP}}+n_{c,\mathrm{FP}}+n_{c,\mathrm{FN}}},$$ (9)

where $n_{c,\mathrm{TP}}$, $n_{c,\mathrm{FP}}$, and $n_{c,\mathrm{FN}}$ are the number of true-positive pixels, false-positive pixels, and false-negative pixels of the class c, respectively, and C is the total number of classes.

**Fig. 4:**
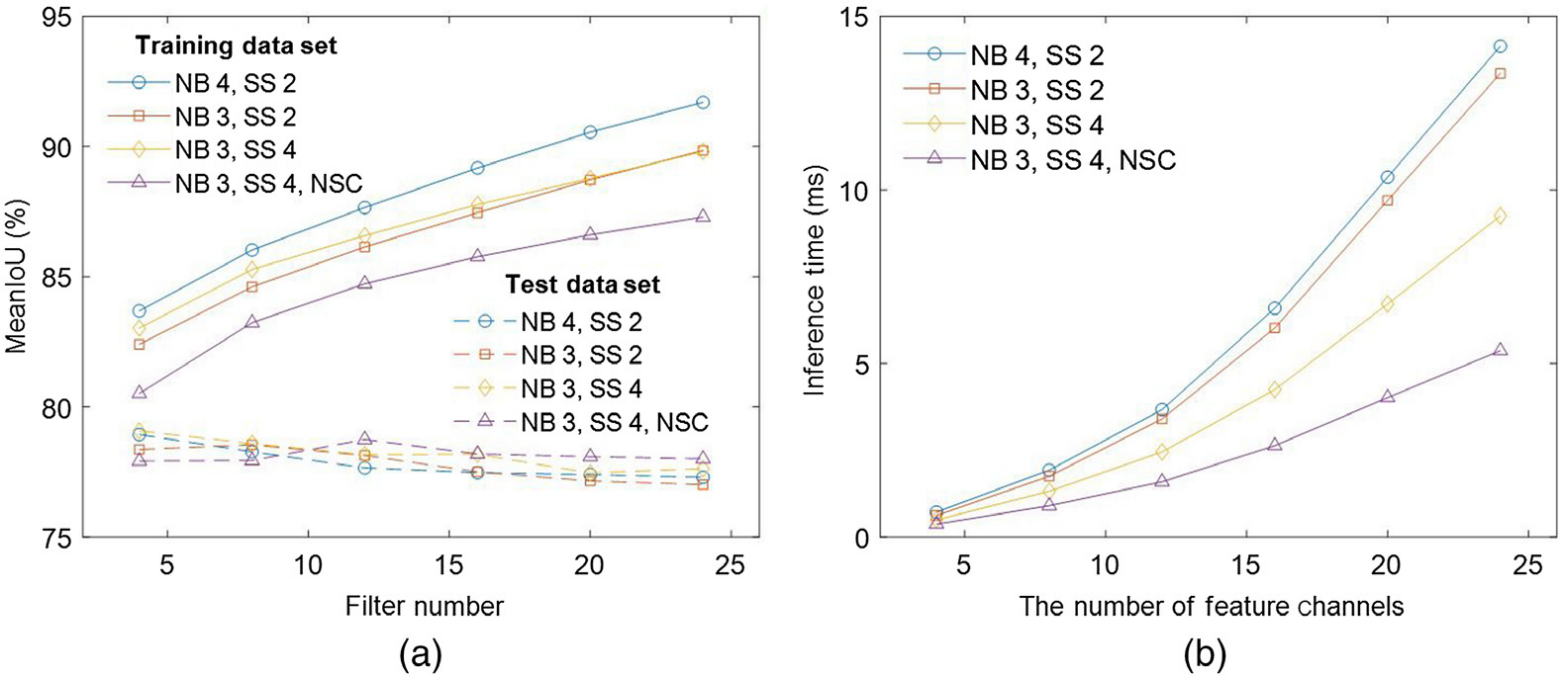
(a) Mean IoU of trained networks on the train and test datasets and (b) inference time on GPU for segmentation of 16 A-scan OCT images of 1×320  pixels. NB, the number of contracting and expanding blocks; SS, sampling size; and NSC, no skip concatenation connection.

Figure 4(a) shows the mean IoU on the train and test datasets as a function of the number of feature channels calculated by networks described in Sec. 2.1. Each CNN architecture was trained five times, and the plots indicate average values. As expected, mean IoU on the train dataset increases with learnable parameters, which increase with the number of contracting and expanding blocks, the number of feature channels, and sampling size, and mean IoU on the test dataset decreases or increases and then decreases with learnable parameters due to overfitting. Also the removal of the skip concatenation connections does not degrade performance distinctively. This could be because our network is not very deep and high-resolution features passed from the contracting path to the expanding path do not advantageously affect the task due to the speckle noise of the images. We achieve the best mean IoU of 79.1% on the test dataset with three contracting and expanding blocks and a sampling size of 4. The inference time of the trained networks on GPU was measured considering real-time axial tremor compensation. The most time-consuming layer is a convolutional layer, so inference time is significantly affected by the number of channels, sampling size, and skip concatenation connection, as shown in Fig. 4(b). The inference time for 16 images of 1×320  pixels is at most 1.6 ms with the optimal number of feature channels for each architecture. Physiological hand tremor has a frequency of 7 to 13 Hz, and its amplitude in the axial direction is around 50  μm.^2^ The speed of physiological hand tremor is approximately calculated as 1  μm/ms assuming a frequency of 10 Hz and linear movement. Therefore, inference time of 1.6 ms is considered reasonably fast for physiological tremor cancellation since other computation and communication delay of our system is around 1.5 ms and image pixel size along the axial direction, the smallest distance we can detect, is 2.7  μm.

Tables 1–3 show the MSE, MUE, and AME of retinal boundary position calculated with an optimal number of feature channels before and after applying the Kalman filter. The MSE, MUE, and AME are defined as follows:

$$\mathrm{MSE}=\frac{1}{N}\sum_{i=1}^{N}{\hat{p}}_{i}-p_{i},$$ (10)

$$\mathrm{MUE}=\frac{1}{N}\sum_{i=1}^{N}|{\hat{p}}_{i}-p_{i}|,$$ (11)

$$\mathrm{AME}=\max_{i=1\ldots N}|{\hat{p}}_{i}-p_{i}|,$$ (12)

where ${\hat{p}}_{i}$ and $p_{i}$ are the estimated and true retinal boundary position of the i’th A-scan images, and N is the total number of A-scan images in the test dataset. The Kalman filtering does not affect MSE distinctively, but it reduces MUE and AME by removing unexpected high-frequency motion of tracked position. Overall, the errors are comparable for the networks presented in the tables, and we selected the last network architecture for our tremor cancellation system because it tracked boundaries more stably with lower MUEs and AMEs. Using the selected parameters for CNN, MSE, MUE, and AME of PR/CH boundary after Kalman filtering were −0.45  pixels (−1.2  μm), 2.48 pixels (6.7  μm), and 16 pixels (43.2  μm), respectively. Relatively larger errors than that of other CNN-based OCT retinal segmentation could be caused by the absence of lateral information and limited image quality obtained by a fiber probe.

**Table 1:** MSE of retinal boundary position (pixels)

Retinal boundary	Convolution filter size and number, and sampling size							
	NB 4, NC 4, SS 2		NB 3, NC 8, SS 2		NB 3, NC 4, SS 4		NB 3, SS 4, NSC	
	CNN	KF	CNN	KF	CNN	KF	CNN	KF
VH/GCL	−1.46	−1.54	**0.22**	**0.14**	−0.72	−0.79	0.67	0.60
GCL/IPL	1.34	1.27	**0.35**	**0.28**	0.96	0.88	1.54	1.48
IPL/INL-OPL	0.46	0.39	−5.37	−5.51	**−0.017**	**−0.09**	−0.068	−0.13
INL-OPL/ONL-ELM	−2.49	−2.57	−1.36	−1.43	−1.45	−1.53	**−1.06**	**−1.12**
ONL-ELM/PR	−1.08	−1.16	−**0.92**	**−1.00**	−**0.92**	−**1.00**	−1.41	−1.49
PR/CH	**−0.23**	**−0.31**	−0.83	−0.90	−0.59	−0.69	−0.38	−0.45

NB, the number of contracting and expanding blocks; NC, the number of feature channels; SS, sampling size; and NSC, no skip concatenation connections.

**Table 2:** MUE of retinal boundary position (pixels).

Retinal boundary	Convolution filter size and number, and sampling size							
	NB 4, NC 4, SS 2		NB 3, NC 8, SS 2		NB 3, NC 4, SS 4		NB 3, NC 12, SS 4, NSC	
	CNN	KF	CNN	KF	CNN	KF	CNN	KF
VH/GCL	3.81	3.01	2.86	**2.04**	3.11	2.50	**2.68**	2.15
GCL/IPL	**4.10**	**3.71**	5.09	4.61	4.24	3.75	4.21	3.79
IPL/INL-OPL	3.82	3.37	8.72	7.94	3.93	3.34	**3.61**	**3.14**
INL-OPL/ONL-ELM	4.06	3.88	3.71	3.45	3.55	3.36	**3.29**	**3.01**
ONL-ELM/PR	2.79	2.43	2.97	2.51	**2.65**	**2.37**	2.93	2.56
PR/CH	2.99	2.56	3.44	2.84	3.20	2.77	**2.88**	**2.48**

**Table 3:** AME of retinal boundary position (pixels).

Retinal boundary	Convolution filter size and number, and sampling size							
	NB 4, NC 4, SS 2		NB 3, NC 8, SS 2		NB3, NC 4, SS 4		NB 3, NC 12, SS 4, NSC	
	CNN	KF	CNN	KF	CNN	KF	CNN	KF
VH/GCL	27.5	19.9	61	21.2	39.5	22.1	**15**	**14.6**
GCL/IPL	21	**15.3**	45	31.3	21	17	**16**	17.2
IPL/INL-OPL	32.5	18.6	61.5	53	48	18.6	**22**	**15.0**
INL-OPL/ONL-ELM	35.5	17.9	53	24.4	**18**	**15.1**	31.5	16.2
ONL-ELM/PR	31	17.1	41	16.8	25.5	15,4	**22**	**14.3**
PR/CH	27	18	48.5	22.9	88	40.6	**24.5**	**16**

### 3.2 Real-Time Ex Vivo Bovine Retinal Boundary Tracking and Tremor Cancellation

We evaluated the retinal boundary tracking and depth targeting performance of the handheld microsurgical instrument guided by CNN using an *ex vivo* bovine retina model.

**Fig. 5:**
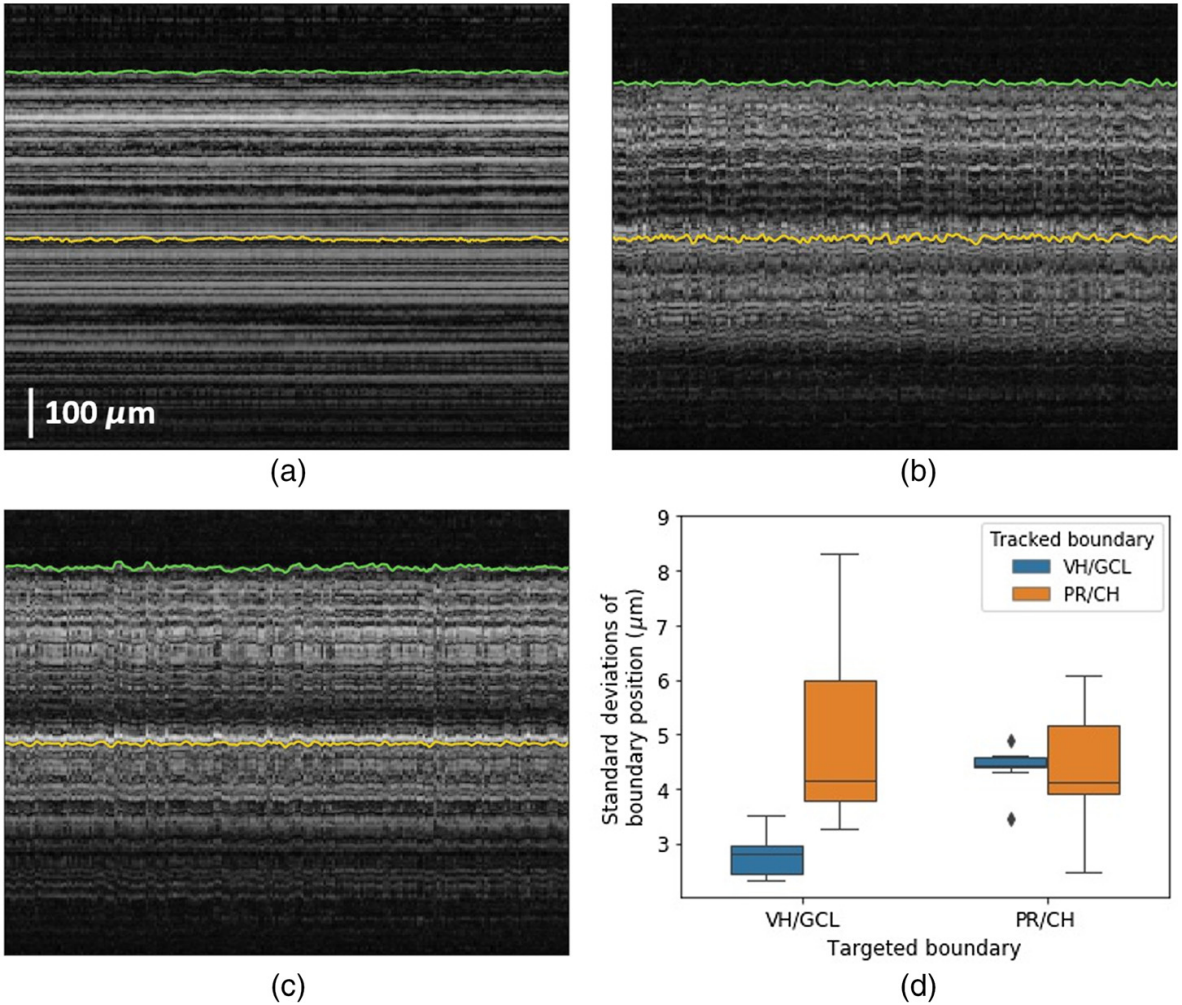
M-scan OCT images of *ex vivo* bovine eyes acquired using (a) a stationary OCT distal sensor and a OCT distal sensor attached to fixed motor activated for (b) VH/GCL boundary targeting and (c) PR/CH boundary targeting. The green and yellow solid lines represent tracked VH/GCL and PR/CH boundaries, respectively. (d) The SDs of tracked boundary positions by an OCT distal sensor attached to a fixed motor during depth targeting.

At first, we produced an estimate of noise for retinal boundary tracking by measuring standard deviations (SDs) of VH/GCL and PR/CH boundary positions using a stationary OCT distal sensor. Figure 5(a) shows the M-scan OCT image for 1 s and tracked boundary positions obtained using the stationary OCT distal sensor. Overall, speckle pattern does not change as expected, but local intensity variations, which could be caused by OCT noise and micro-oscillations inside a sample, induce small fluctuations of tracked retinal boundaries. Therefore, although the SDs of boundary positions are supposed to be zero because the distance between the retina and the OCT distal sensor does not vary, the mean and SD of the SDs acquired from 13 trials of 5 eyes are 2.83±0.69  μm (1.04±0.26  pixel) for VH/GCL boundary and 3.09±0.92  μm (1.14±0.34  pixel) for PR/CH boundary.

Depth targeting system noise was then evaluated using a piezoelectric motor fixed to a stationary stage. The motor was integrated with an OCT distal sensor attached needle and activated for depth targeting of the needle. Ideally, the motor should be stabilized when the needle reaches a target depth since both the motor and the sample are stationary. However, due to retinal boundary tracking noise and control error, the motor kept working actively as shown in Figs. 5(b) and 5(c). Figures 5(b) and 5(c) show M-scan OCT images for 1 s, when VH/CGL boundary and PR/CH boundary are targeted, respectively. The SDs of VH/GCL and PR/CH boundary positions during depth targeting were measured with 13 trials of 5 eyes and shown in Fig. 5(d). The mean and SD of the SDs of VH/GCL and PR/CH boundary positions are 2.75±0.35  μm (1.02±0.13  pixel) and 4.8±1.46  μm (1.78±0.54  pixel), respectively, when the VH/CGL boundary is targeted. When the PR/CH boundary is targeted, the mean and SD of the SDs of VH/GCL and PR/CH boundary positions are 4.41±0.31  μm (1.63±0.12  pixel) and 4.28±1.02  μm (1.58±0.38  pixel), respectively. Theoretically, the speckle pattern does not change with axial motion only, so the overall speckle pattern does not change significantly except shifts in the axial direction. However, local intensity variations of the speckle pattern increase with axial motion because the OCT sensing beam is not perfectly perpendicular to the retina surface and axial motion could induce slight transverse motion. Moreover, since the sensing beam is focused on the retina, the axial motion changes the integration volume inside the retina, which also could increase local intensity variations. Therefore, PR/CH boundary tracking, which has a larger tracking error, is degraded more by the intensity variations and shows larger SDs than that of VH/GCL boundary tracking.

**Fig. 6:**
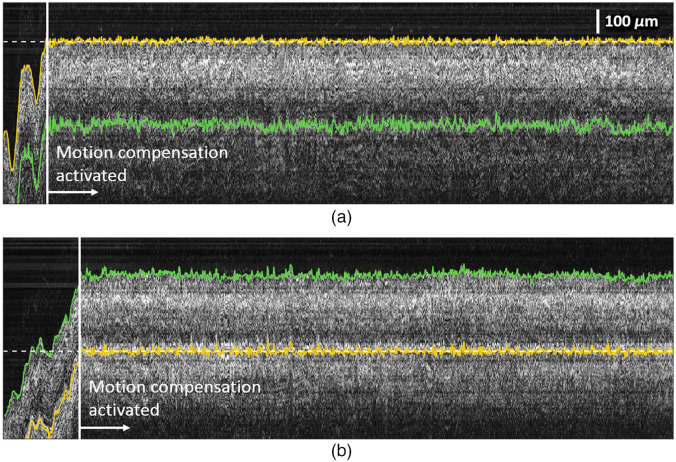
M-scan OCT images of *ex vivo* bovine eyes with and without tremor cancellation (a) when a boundary between VH and GCL is targeted and (b) when a boundary between PR and CH is targeted. The yellow and green solid lines are targeted boundary and untargeted another boundary, respectively. The dashed lines represent target depth, and white vertical lines indicate the moment when motion compensation has been activated.

**Fig. 7:**
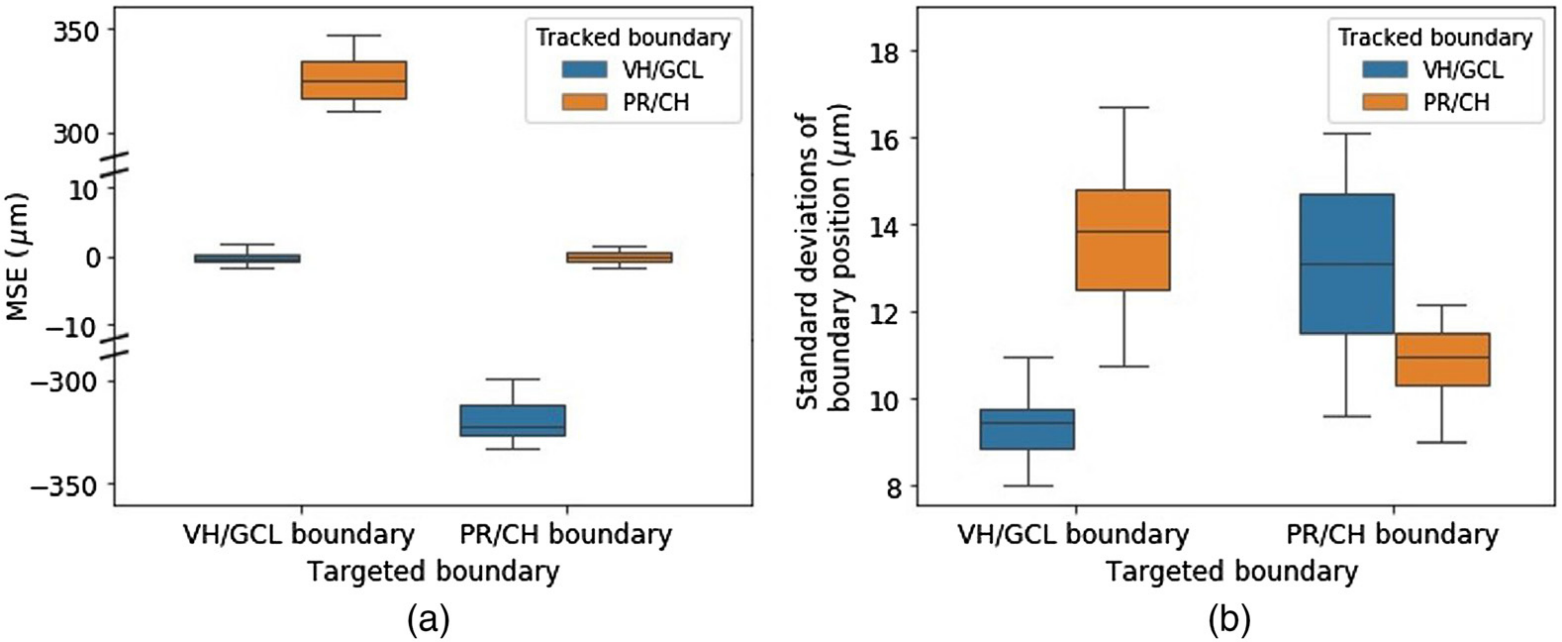
Box plots of (a) MSEs and (b) SDs of the VH/GCL and PR/CH boundary positions during VH/GCL boundary targeting and PR/CH boundary targeting.

Tremor compensation and depth targeting performance were evaluated for a handheld microsurgical instrument. The microsurgical instrument was held by a free-hand and proceeded toward the retina until automatic depth targeting was activated. We used a tremor compensation algorithm we developed earlier and more details can be found in our previous work.^13^ A VH/GCL boundary, as well as a PR/CH layer boundary, were tracked, and one of them was used for depth targeting. We performed 12 trials of depth targeting each for VH/CGL and PR/CH boundaries using 5 eyes. Figure 6 shows the M-scan OCT images of the bovine retina obtained with and without tremor compensation for ∼13 s. In Fig. 6(a), the VH/GCL boundary (yellow line) was used for depth targeting, and its target depth represented by the dashed line was set to 700  μm away from a fiber probe end. Similarly, in Fig. 6(b), the PR/CH boundary (yellow line) was targeted, and its target depth was set to 1000  μm. The green solid lines are untargeted boundaries (VH/GCL or PR/CH), and the white vertical lines indicate the moment when motion compensation has been activated. The left side of the vertical line with a highly irregular boundary profile represents duration without the tremor compensation, however, once the tremor compensation has been activated (right side of the vertical line), the targeted boundary becomes flat and fixed around the target depth indicating that the motion compensation is working effectively. As expected, when VH/GCL or PR/CH boundary is targeted, the axial variation of another boundary positions increases, and it is quantitatively verified by comparing the MSEs and SDs of the tracked boundary positions for each trial. Here the MSE is defined as follows:

MSE=1N∑i=1Np^i−ptarget, (13)

where ${\hat{p}}_{i}$ and $p_{\text{target}}$ are the estimated and targeted retinal boundary position, respectively, and N is the total number of A-scan images of each trial. In Fig. 7(a), the mean and SD of the MSEs of targeted boundaries are −0.15±1.02  μm for the VH/CGL boundary and −0.11±0.96  μm for the PR/CH boundary, and the mean and SD of the MSEs of untargeted boundaries are −319.52±10.13  μm for the VH/GCL boundary and 325.72±11.35  μm for the PR/CH boundary. Untargeted boundaries have almost ten times larger variations of MSEs than that of targeted boundaries because of retinal thickness variations between different eyes and different areas, and this result supports the necessity of PR/CH boundary tracking rather than just surface tracking for accurate subretinal injection guidance. The mean and SD of SDs of targeted boundaries are 9.42±0.80  μm for the VH/GCL boundary and 10.8±0.90  μm for the PR/CH boundary. The axial motion mostly from the hand tremor, which includes low-frequency draft in the order of hundreds of micrometers and physiological tremor in the order of tens of micrometers, is reduced significantly. The residual variations are caused by the boundary tracking error and the time delay between the signal processing and motor control. The slightly better performance of the VH/GCL boundary targeting could be explained by the more accurate tracking of the VH/GCL boundary as shown in Sec. 3.1. The mean and SD of the SDs of untargeted boundaries are 13.03±1.96  μm for VH/GCL boundary and 13.67±1.79  μm for the PR/CH boundary. Retinal thickness variations within an eye increased the SDs of the untargeted boundaries due to lateral motion of hand tremor.

**Fig. 8:**
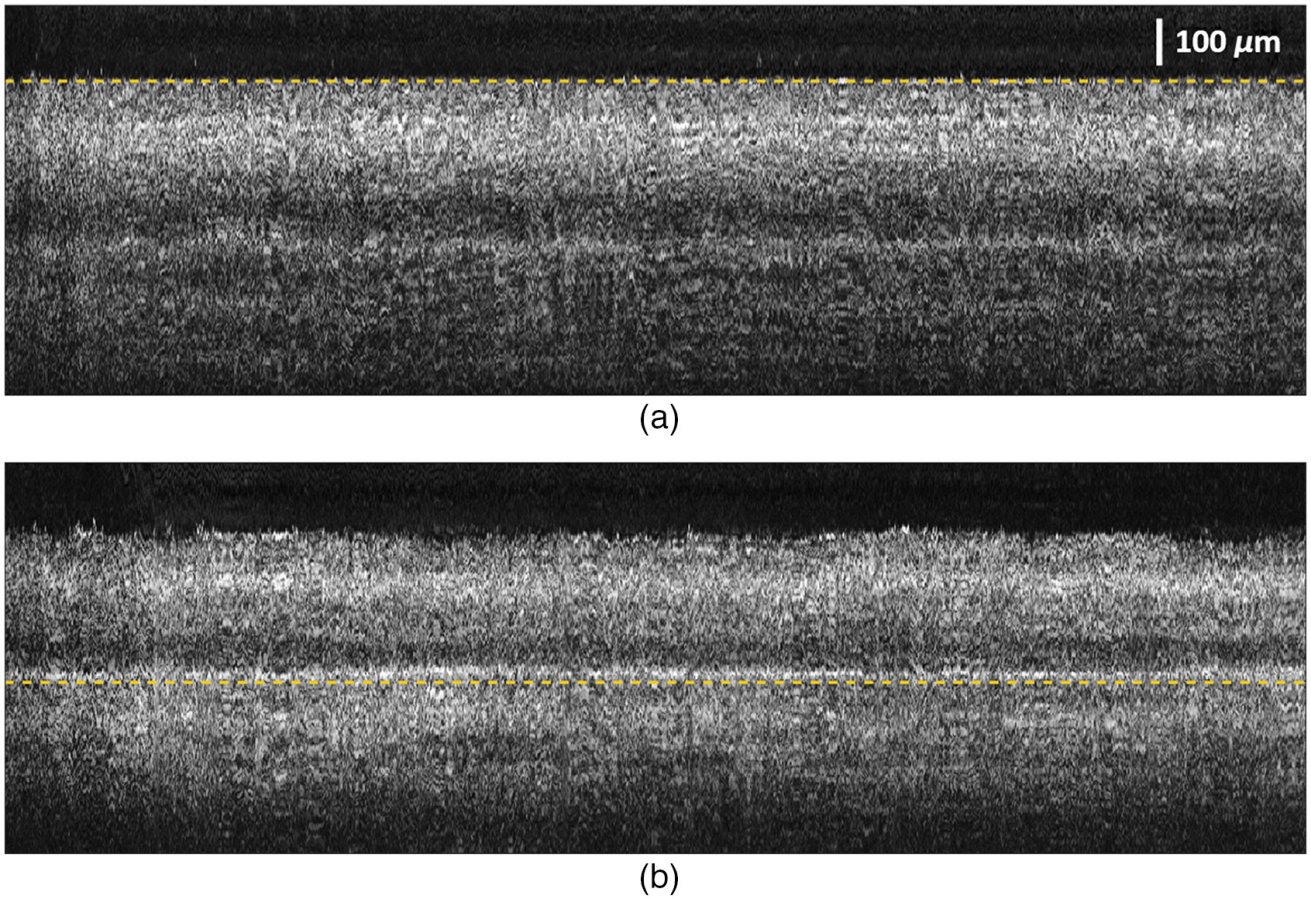
M-scan OCT images of *ex vivo* bovine eyes when each A-scan image is aligned to the target boundaries: (a) the VH/GCL boundary and (b) the PR/CH boundary.

It is difficult to obtain a precise ground-truth segmentation label from our M-scan OCT images (Fig. 6) because of high-frequency longitudinal fluctuations and speckle noise and thus to evaluate the accuracy of the tracked boundary positions quantitatively. Nevertheless, we can assess it visually by checking how flat and smooth the targeted retinal boundary is when each A-scan image is aligned to the tracked boundary position. The more accurate boundary tracking brings the flatter and smoother target boundaries in the aligned M-scan images. Figures 8(a) and 8(b) show the aligned M-scan images to the target boundaries, the VH/GCL boundary and the PR/CH boundary, represented by yellow dashed lines. High-frequency fluctuations shown in Fig. 6 were significantly reduced in the regions around the targeted boundaries, and we could infer that retinal boundary tracking works effectively.

## 4 Conclusion

In this paper, we presented real-time A-scan-based CNN segmentation and automatic retinal boundary targeting for handheld subretinal injector guidance. A-scan retinal OCT images are segmented using a simplified 1D U-net, and the Kalman filter reduces retinal boundary tracking error by combining boundary position measurement and velocity measurement. We achieve the MUE of around 3 pixels (8.1  μm) using an *ex vivo* bovine retina model. GPU parallel computing allows real-time inference (∼1.6  ms) and thus real-time retinal boundary tracking. The MSE between target depth and target boundary position of the depth targeting experiment is −0.15 and 0.11  μm for the VH/GCL and the PR/CH boundary, respectively. Involuntary tremors, which include low-frequency draft in the order of hundreds of micrometers and physiological tremor in the order of tens of micrometers, are reduced significantly, and the SDs of target boundary positions are 9.42  μm for the VH/GCL boundary and 10.8  μm for the PR/CH boundary. Our networks currently work only for normal bovine retina, but in the future, we will expand its utility to diseased retina having irregular morphology by including diseased retinal images into our train dataset. We also plan to perform *ex vivo* and *in vivo* studies of subretinal injection using our system to validate its clinical applicability.

## References

[1] Peng Y. Tang L. Zhou Y., “Subretinal injection: a review on the novel route of therapeutic delivery for vitreoretinal diseases,” Ophthal. Res. 58(4), 217–226 (2017). OPRSAQ 0030-3747 10.1159/000479157 28858866

[2] Hotraphinyo L. F. Riviere C. N., “Three-dimensional accuracy assessment of eye surgeons,” in Conf. Proc. 23rd Annu. Int. Conf. IEEE Eng. Med. and Biol. Soc., Vol. 4, pp. 3458–3461 (2001). 10.1109/IEMBS.2001.1019574

[3] Singh S. P. N. Riviere C. N., “Physiological tremor amplitude during retinal microsurgery,” in Proc. IEEE 28th Annu. Northeast Bioeng. Conf. (IEEE Cat. No. 02CH37342), pp. 171–172 (2002). 10.1109/NEBC.2002.999520

[4] Fercher A. F. et al., “Optical coherence tomography: principles and applications,” Rep. Prog. Phys. 66, 239–303 (2003). RPPHAG 0034-4885 10.1088/0034-4885/66/2/204

[5] Kang J. U. et al., “Real-time three-dimensional Fourier-domain optical coherence tomography video image guided microsurgeries,” J. Biomed. Opt. 17(8), 081403 (2012). JBOPFO 1083-3668 10.1117/1.JBO.17.8.081403 23224164 PMC3381017

[r6] Zhang K. Kang J. U., “Real-time intraoperative 4D full-range FD-OCT based on the dual graphics processing units architecture for microsurgery guidance,” Biomed. Opt. Express 2, 764–770 (2011). BOEICL 2156-7085 10.1364/BOE.2.000764 21483601 PMC3072119

[r7] Draelos M. et al., “Optical coherence tomography guided robotic needle insertion for deep anterior lamellar keratoplasty,” IEEE Trans. Biomed. Eng. 67(7), 2073–2083 (2020). IEBEAX 0018-9294 10.1109/TBME.2019.2954505 31751219 PMC7365552

[r8] Zhou M. et al., “Towards robotic-assisted subretinal injection: a hybrid parallel-serial robot system design and preliminary evaluation,” IEEE Trans. Ind. Electron. 67(8), 6617–6628 (2020). 10.1109/TIE.2019.2937041

[9] Sommersperger M. et al., “Real-time tool to layer distance estimation for robotic subretinal injection using intraoperative 4D OCT,” Biomed. Opt. Express 12, 1085–1104 (2021). BOEICL 2156-7085 10.1364/BOE.415477 33680560 PMC7901333

[10] Song C. et al., “Fiber-optic OCT sensor guided “smart” micro-forceps for microsurgery,” Biomed. Opt. Express 4, 1045–1050 (2013). BOEICL 2156-7085 10.1364/BOE.4.001045 23847730 PMC3704086

[r11] Cheon G. W. et al., “Accurate real-time depth control for CP-SSOCT distal sensor based handheld microsurgery tools,” Biomed. Opt. Express 6, 1942–1953 (2015). BOEICL 2156-7085 10.1364/BOE.6.001942 26137393 PMC4467719

[r12] Cheon G. W. et al., “Motorized microforceps with active motion guidance based on common-path SSOCT for epiretinal membranectomy,” IEEE/ASME Trans. Mechatron. 22(6), 2440–2448 (2017). IATEFW 1083-4435 10.1109/TMECH.2017.2749384 PMC5881930 29628753

[13] Kang J. U. Cheon G. W., “Demonstration of subretinal injection using common-path swept source OCT guided microinjector,” Appl. Sci. 8, 1287 (2018). 10.3390/app8081287

[14] Yazdanpanah A. et al., “Intra-retinal layer segmentation in optical coherence tomography using an active contour approach,” Lect. Notes Comput. Sci. 5762, 649–656 (2009). LNCSD9 0302-9743 10.1007/978-3-642-04271-3_79 20426167

[15] González-López A. et al. et al., “Robust segmentation of retinal layers in optical coherence tomography images based on a multistage active contour model,” Heliyon 5(2), e01271 (2019). 10.1016/j.heliyon.2019.e01271 30891515 PMC6401526

[16] Li K. et al., “Optimal surface segmentation in volumetric images-a graph-theoretic approach,” IEEE Trans. Pattern Anal. Mach. Intell. 28(1), 119–134 (2006). ITPIDJ 0162-8828 10.1109/TPAMI.2006.19 16402624 PMC2646122

[r17] Garvin M. K. et al., “Automated 3-D intraretinal layer segmentation of macular spectral-domain optical coherence tomography images,” IEEE Trans. Med. Imaging 28(9), 1436–1447 (2009). ITMID4 0278-0062 10.1109/TMI.2009.2016958 19278927 PMC2911837

[18] Hu Z. et al., “Multiple layer segmentation and analysis in three-dimensional spectral-domain optical coherence tomography volume scans,” J. Biomed. Opt. 18(7), 076006 (2013). JBOPFO 1083-3668 10.1117/1.JBO.18.7.076006 23843084

[19] Chiu S. J. et al., “Automatic segmentation of seven retinal layers in SDOCT images congruent with expert manual segmentation,” Opt. Express 18, 19413–19428 (2010). OPEXFF 1094-4087 10.1364/OE.18.019413 20940837 PMC3408910

[20] Tian J. et al., “Real-time automatic segmentation of optical coherence tomography volume data of the macular region,” PLoS One 10(8), e0133908 (2015). POLNCL 1932-6203 10.1371/journal.pone.0133908 26258430 PMC4530974

[21] Roy A. G. et al., “ReLayNet: retinal layer and fluid segmentation of macular optical coherence tomography using fully convolutional networks,” Biomed. Opt. Express 8, 3627–3642 (2017). BOEICL 2156-7085 10.1364/BOE.8.003627 28856040 PMC5560830

[r22] Shah A. et al., “Multiple surface segmentation using convolution neural nets: application to retinal layer segmentation in OCT images,” Biomed. Opt. Express 9, 4509–4526 (2018). BOEICL 2156-7085 10.1364/BOE.9.004509 30615698 PMC6157759

[r23] Devalla S. K. et al., “DRUNET: a dilated-residual U-Net deep learning network to segment optic nerve head tissues in optical coherence tomography images,” Biomed. Opt. Express 9, 3244–3265 (2018). BOEICL 2156-7085 10.1364/BOE.9.003244 29984096 PMC6033560

[24] Borkovkina S. et al., “Real-time retinal layer segmentation of OCT volumes with GPU accelerated inferencing using a compressed, low-latency neural network,” Biomed. Opt. Express 11, 3968–3984 (2020). BOEICL 2156-7085 10.1364/BOE.395279 33014579 PMC7510892

[25] Ronneberger O. Fischer P. Brox T., “U-Net: convolutional networks for biomedical image segmentation,” Lect. Notes Comput. Sci. 9351, 234–241 (2015). LNCSD9 0302-9743 10.1007/978-3-319-24574-4_28

[26] Venhuizen F. G. et al., “Robust total retina thickness segmentation in optical coherence tomography images using convolutional neural networks,” Biomed. Opt. Express 8, 3292–3316 (2017). BOEICL 2156-7085 10.1364/BOE.8.003292 28717568 PMC5508829

[27] Welch G. Bishop G., “An introduction to the Kalman filter,” Siggraph Course 8, 1–16 (2006).

[28] Lee S. et al., “Common-path all-fiber optical coherence tomography probe based on high-index elliptical epoxy-lensed fiber,” Opt. Eng. 58(2), 026116 (2019). 10.1117/1.OE.58.2.026116

